# The role of exogenous epidermal growth factor on Ki-67 proliferation marker expression in the submandibular salivary gland of albino rats receiving doxorubicin

**DOI:** 10.12688/f1000research.27186.1

**Published:** 2020-12-03

**Authors:** Mohamed Mansy, Malak Soliman, Rabab Mubarak, Mohamed Shamel

**Affiliations:** 1 Department of Oral Biology, Jazan University, Jazan, Saudi Arabia; 2 Department of Oral Biology, Cairo University, Cairo, Egypt; 3 Department of Oral Biology, Cairo University,Vice Dean of faculty of Dentistry, Deraya University, Cairo, Egypt; 4 Department of Oral Biology, The British University in Egypt, Shorouk city, Egypt

**Keywords:** Epidermal growth factor, Ki-67, Doxorubicin, submandibular salivary gland

## Abstract

**Background:** This study was conducted to evaluate the role of exogenous epidermal growth factor (EGF) injection on the Ki-67 immuno-expression in submandibular salivary gland tissue of rats receiving doxorubicin (DXR).

**Methods:** A total of 21 two-month-old male albino rats, of 200 g body weight, were divided into three groups: control group; DXR group, the rats received 20 mg/kg body weight DXR as a single intra peritoneal injection; DXR+EGF group, the rats received the same dose of DXR and on the next day they were injected intraperitoneally with 10 µg/kg body weight of EGF daily for one week. Histological sections and immunohistochemical expression of Ki67 sections were examined using a ZEISS Primo Star light microscopy and images taken using Tucsen IS 1000 10.0MP Camera.

**Results:** Ki-67 expression was significantly increased in submandibular salivary glands of rats after DXR injection. However, Ki-67 expression in the glandular tissue was restored to normal levels after EGF injection.

**Conclusions:** EGF preserved glandular architecture after DXR injection and maintained Ki-67 immune-expression within the glandular tissue near to the normal level.

## Introduction

Ki-67 is a heavy protein of 395 kDa weight. It is a proliferation marker which is highly expressed in various tumors, and has been used for investigations of many cancer types. Ki-67 is controlled by proteolytic pathways and has similar essential properties with other proteins known to regulate the cell cycle ( Hofmann & Bucher, 1995). Furthermore, Ki-67 is an important protein for cell division as antisense nucleotides of Ki-67 will stop the division, and it is a vital factor in the formation of ribosomes ( Schluter *et al*., 1993). This is reinforced by the conclusion that Ki-67 immuno-expression associates with the rate of protein production and function of ribosomes ( Plaat *et al*., 1999).

Proliferation marker evaluation is of high value in pathological diagnosis and prognosis. It has been reported that Ki-67 has a prognostic character for many forms of malignant tumors, such as lymphomas, breast, prostate and colorectal cancers ( Tretiakova *et al*., 2016). Ki-67 is a protein formed during active phases of the cell mitotic cycle, but is not present in resting cells. Therefore, its expression is used as an assessment tool for tissue proliferation ( Faur *et al*., 2015).

The division activity measured by Ki-67 has been reported in previous studies, and has a great prognostic significance in different forms of malignancies ( Yerushalmi *et al*., 2010). Ki-67 is a protein linked with cell production and is noticeable in all active phases of the cell cycle cresting at G2 and persisting at low levels after cell cycle exit. It then becomes undetectable in senescent cells ( Sobecki *et al*., 2017).

Epidermal growth factor (EGF) could motivate the production and differentiation of epidermal cells and assist skin renewal and wound healing. The therapeutic effect of EGF in the treatment of thermal injuries is not only confined to rapid activation of the healing process and a decrease in tissue damage, but also decreases the size of the affected area and reduces hyperergic inflammation. EGF has demonstrated its efficacy in thermic injury by stimulating wound healing and decreasing the possibility of purulent septic complication and tissue damage. This process might also involve modulation of the immune system status ( Osikov *et al*., 2014; Parment *et al*., 2007).

Doxorubicin (DXR) is an important drug for leukemia, Hodgkin's lymphoma and bladder, breast, stomach, lung and ovaries cancer, treatment during chemotherapy ( Bielack *et al*., 1996). Jensen *et al*. (2002) studied the consequence of chemotherapy on the salivary gland with different solid and hematological tumors. Apart from xerostomia, 50% of the salivary glands of the patients showed ductal dilatation, cyst formation, degenerated acini and inflammatory cell presence. These degenerated salivary glands were markedly detected less than 2 weeks after chemotherapy. DXR motivates reactive oxygen species (ROS) synthesis and depolarizes the membrane potential of the mitochondria. Both excessive ROS synthesis and mitochondrial membrane damage are very important causes of cellular injury ( Ghosh *et al*., 2011).

This study was conducted to evaluate the role of exogenous EGF injection on the Ki-67 immuno-expression in submandibular salivary gland tissue of rats receiving DXR. Rats were used in this study because they have biology similar to humans and therefore can be a model for human carcinogenicity and recovery.

## Methods

### Animals

This study protocol and the animal care and experimental procedures were approved by the Ethics Committee, Faculty of Dentistry, Cairo University, Egypt (#415). All efforts were made to ameliorate any suffering of the animals by adopting the OECD 423 test guidelines, and all applicable international, national, and/or institutional guidelines for the care and use of animals were followed.

In total, 21 male adult albino rats, two months old, pathogen free, with an average weight of 200 gm were used. The animals were obtained from and housed in the Animal house, Faculty of Medicine, Cairo University. Sample size calculation was performed using G*Power version 3.1.9.2 (University Kiel, Germany) ( Faul *et al*., 2009). The effect size was 0.95 using α level of 0.05 and β level of 0.05, i.e., power = 95%; the estimated sample size (n) was a total of 21 samples for three groups.

The animals were housed in a controlled environment (temperature 25±2°C, humidity 70–80% and 12hr dark/light cycle) and had free access to food and water. The animals were fed a natural diet and water *ad libitum* throughout the whole experiment. The rats were acclimatized to their cages for 1 week.

All 21 rats were given a number (1–21) using a marker pen, then randomized by putting the numbers in an envelope and dividing them into three groups according to the numbers which were taken from the envelope.

The three groups were as follows:

- control group, the rats were kept on a normal diet and did not receive DXR or EGF;
- DXR group, the rats received 20 mg/kg body weight DXR as a single intra-peritoneal injection ( Ayla *et al*., 2011);
- DXR+EGF group, the rats received the same intraperitoneal dose of DXR (20 mg/kg body weight) and on the next day they were injected intraperitoneally with 10µg/kg body weight of EGF daily for one week ( Ohlsson *et al*., 1997).

The rats were injected every morning at 9 am in the animal house laboratory of Faculty of Medicine, Cairo university.

### Procedure

This study was performed to detect changes in the salivary glands after doxorubicin injection and the role of EGF, if any, in reversing any negative changes appearing in the glands. Therefore, histological sections as well as Ki67 immuno expression were used to detect these changes.

The rats were sacrificed by euthanization by CO _2_ asphyxiation followed by cervical dislocation when the experiment finished after 1 week.

Submandibular salivary glands of both sides were dissected out and preparation of specimens for staining procedure was done as follows.

After the glands were excised, those of the right side were fixed immediately in 10% neutral buffered formalin. Then, the specimens were washed properly under running water, dehydrated through ascending concentrations of alcohol and transferred to xylene to clear the specimens from alcohol. Then, the glands were embedded in paraffin wax and mounted in the center of the paraffin wax blocks. Sections from paraffin embedded tissues blocks were cut into sections 5-µm thick and mounted on glass slides for histological examination using Samples were processed for regular histopathological examination using H&E stain. Other sections were stained with immuno-peroxidase for immunohistochemical detection of Ki-67 in the glandular tissue using staining reaction containing anti-Ki-67 antibodies (Santa Cruz Biotechnology catalogue # sc-23900).

The selected sections were studied by ZEISS Primo Star light microscopy and images taken using Tucsen IS 1000 10.0MP Camera in the Oral Biology Lab, Faculty of Dentistry, Cairo University.

The scoring of the staining reaction of the immunohistochemical parameter (presence of Ki-67) of the different groups was as follows: (-) negative reactivity and (+) positive reactivity.

### Image analysis

Image analysis was performed using a computer system software Leica Quin 500 (LEICA Imaging Systems Ltd, Cambridge, England) to assess the area percentage of the immunostaining within the tissues.

The image analyzer was first calibrated automatically to convert the measurement units (pixels) produced by the image analyzer program into actual micrometer units. Measurement of the area of percentage positive cells was done as previously described ( Shi *et al*., 2007). Briefly, the percentage of positive cells was recorded as an area and area percentage within a standard measuring frame of area 114,342.9 mm ^2^ per 10 fields from different slides. This was done at a magnification of 400X by the light microscope transmitted to the monitor. Areas containing the most uniformly stained tissues were chosen for evaluation. These areas were disguised by blue binary color which could be measured by the computer system. Images were manually corrected for brightness and contrast. Colour thresholding was then performed automatically after which pictures were converted to RGB stack type. Masking of the brown cytokeratin, immuno-stain was performed by blue colour where any brown stain of any intensity was considered positive whereas the background grey stain was considered negative. Area fraction was then calculated automatically representing the area percentage of immune positive cells to the total area of the microscopic field.

### Data analysis

Image analysis data representing experimental values of Ki-67 immunostain were given as mean and standard deviation. ANOVA (ONEWAY ANOVA test, n=10, P <0.05) was used to compare the mean area percent of Ki-67 immuno-expression among the specimens of different groups. Tukey’s Multiple Comparison Test (Post Hoc Tukey HSD) was performed to calculate a pair-wise comparison between each group. SPSS 25.0 for Windows (SPSS Inc., Chicago, IL, USA) was used for analysis.

## Results

### Histological examination

**Figure 1.**
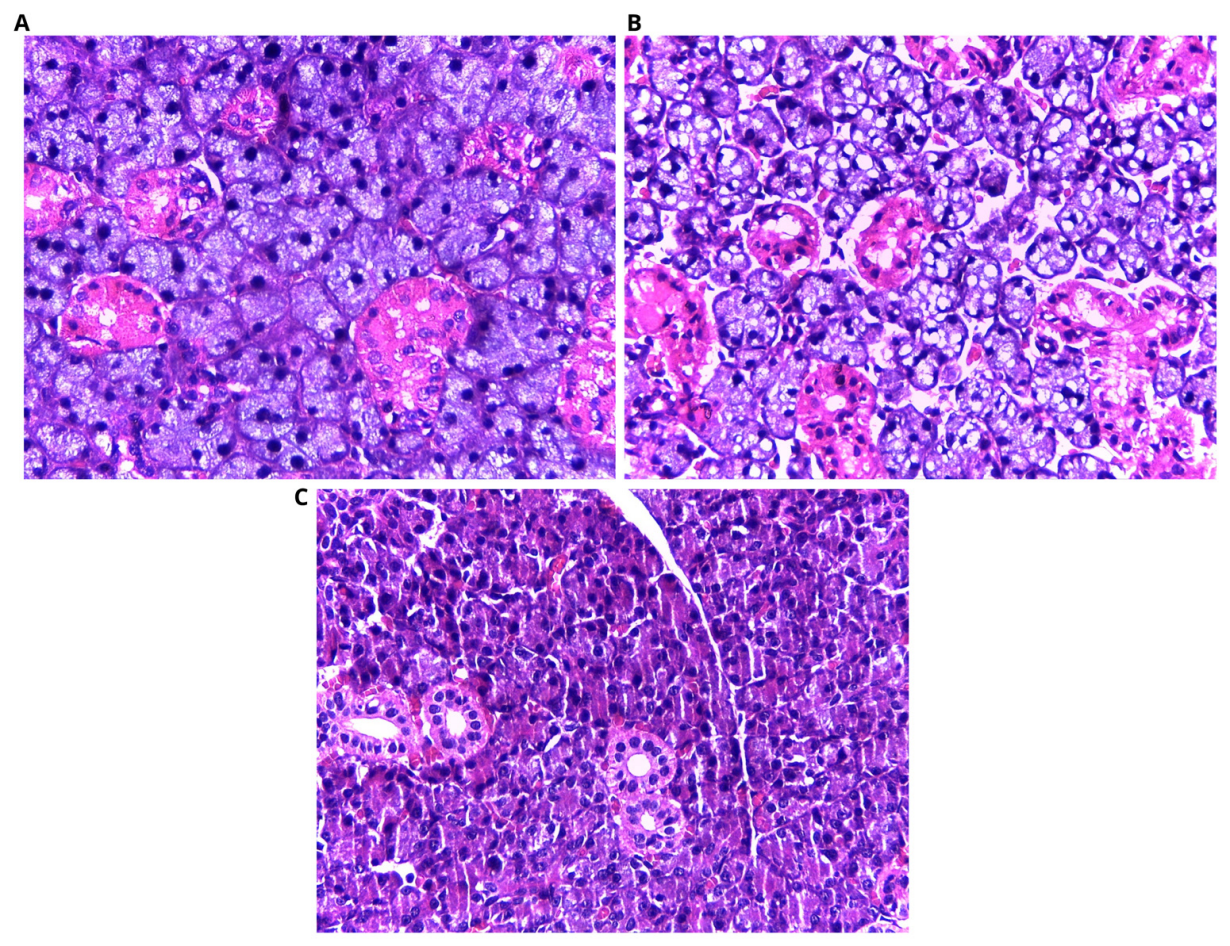
Photomicrographs showing histology of salivary glands. ( **A**) Control group, regular gland architecture; ( **B**) DXR group, degenerated acini with multiple vacuoles extravasated red blood cells (RBCs) in between acini and ducts; ( **C**) DXR+EGF group, well aligned acini together with blood vessels engorged with RBCs in association with the striated ducts exhibiting good cell alignment and minute areas of small vesicles. H&E staining, orig. mag. X400.

Histological examination of the submandibular salivary gland of rats of control group (group I) revealed its main structural components was composed of parenchymal tissue supported by connective tissue stroma ( Figure 1A). Histopathological sections of DXR group (group II) showed several pathological changes. The secretory acini appeared with massive cytoplasmic vacuolization, and deformation in the acini and loss of normal cellular orientation were frequently encountered. A clear space separated the parenchymal elements, which might be an index of interstitial edema and extravasated red blood cells (RBCs) in between acini and ducts ( Figure 1B).

Comparing with the histopathological results of DXR group, the DXR+EGF group (group III) sections showed great enhancement in the structural features of the glands. Little evidence of inflammatory condition was present. On the other hand, many blood vessels engorged with RBCs were found in close relation with the striated ducts. Moreover, a rich vascular network was found in association to the excretory ducts ( Figure 1C).

### Ki-67 immunoreactivity

**Figure 2.**
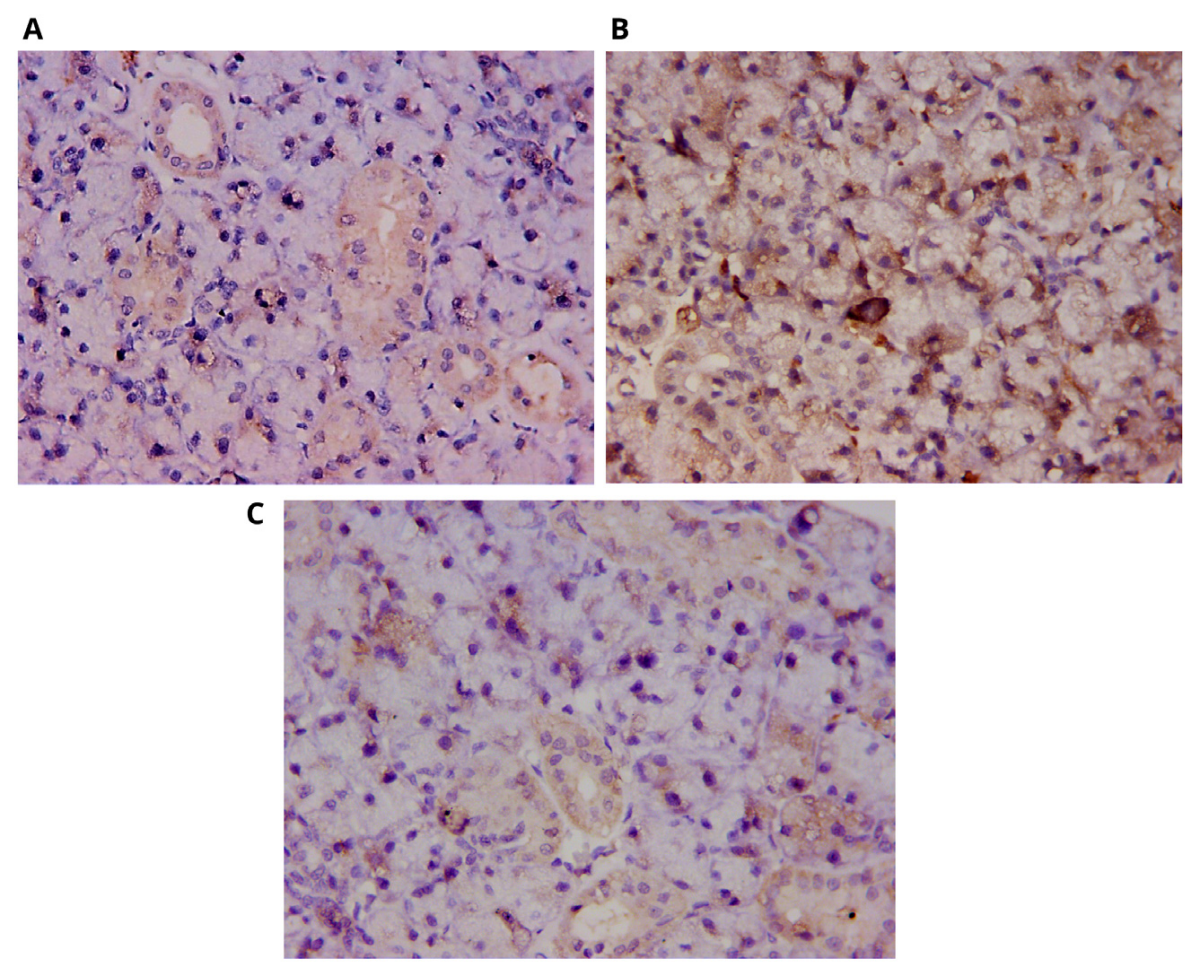
Photomicrographs showing Ki-67 immunoreactivity. ( **A**) Control group, positive reaction to Ki-67 protein in the secretory acini and the wall of the blood vessels; ( **B**) DXR group, increased reactivity of Ki-67 in the secretory acini together with the nuclear and perinuclear reactivities; ( **C**) DXR+EGF group, decreased Ki-67 immunoreactivity in the acini. DAB, orig. mag. X400.

The control group sections showed positive cytoplasmic immunoreactivity for Ki-67 protein in the parenchymal tissue of the glands, which appeared more distinctive in the duct system. Scattered nuclear reactivities were identified for the protein antigen. A few localized focal areas in the secretory acini as well as the endothelial cells of blood vessels expressed the proliferation antigen at higher intensity ( Figure 2A). On the other hand, the immunohistochemical findings of the DXR group corroborated with the histological results; Ki-67 protein was localized in the DXR group sections differently to the control group. DXR was found to increase the immuno-expression of Ki-67 protein in the submandibular salivary glands of rats particularly the secretory terminal portions. Wider areas of the acini expressed the antigen and with greater intensity. The expression of the proliferation antigen appeared foamy, probably due to the vacuolar degeneration affecting the glandular tissue. Scattered nuclear and perinuclear reactivities were identified for the protein antigen in this group ( Figure 2B).

**Figure 3.**
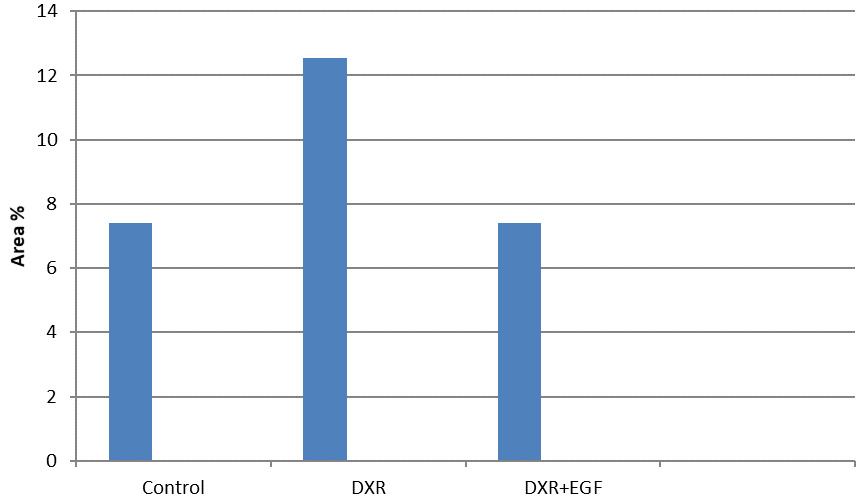
Area percent of Ki-67 reactivity in the submandibular salivary glands of all groups.

The differences between the control and DXRs groups were statistically significant, as there was a significant increase in the mean area percent of Ki-67 immuno-expression found in DXR group in contrast with the control group (p<0.01; Figure 3).

The tissue sections of the DXR+EGF group revealed a remarkable decrease in Ki-67 antigen expression in the acinar cells in comparison to the DXR group. Conversely a slight increase in immunoreactivity to the proliferation antigen appeared in the ductal profiles. Nevertheless, the immunoreactivity seemed to be very similar to that of the control group ( Figure 2C).

Post Hoc Tukey HSD test comparing between the groups showed that the difference between the control group and DXR+EGF group was not significant (p>0.05). However, a significant increase was recorded between the DXR and control groups, and a significant decrease was recorded between DXR+EGF and DXR groups (p<0.01; Table 1 and Table 2).

**Table 1.** Area percentage of Ki-67 immuno-expression in different groups.

	Control	DXR	DXR+EGF	P- value
Minimum	5.486	9.958	4.42	0.001
Median	6.96	12.13	5.62	
Maximum	8.731	15.118	9.67	
Mean	7.399	12.538	6.277	
Standard Deviation	1.118	1.661	2.243	
Standard Error	0.5	0.743	0.916	

**Table 2.** Tukey’s Post Hoc Test for Ki-67 immuno-expression in different groups.

Tukey’s multiple comparison	Significance (P>0.05)	Summary
Control vs DXR	0.003	Significant
Control vs DXR+EGF	0.778	Not significant
DXR vs DXR+EGF	0.0001	Significant

## Discussion

In this study, the systemic injection of DXR in rats resulted in pathological structural alterations within the submandibular salivary gland tissue. Atrophied and degenerated acini with multiple cytoplasmic vacuolization were detected. The chemical composition of DXR leads to creation of free radicals and the stimulation of oxidative stress, which is considered the main factor of cellular damage ( Saad *et al*., 2001). DXR also initiates an inequity between free oxygen radicals and antioxidants. This disturbs the oxidant–antioxidant system, leading to tissue damage that is manifested by lipid peroxidation and protein oxidation within the tissue ( Karaman *et al*., 2006).

Mitochondrial degeneration possibly is the primary cause of the intracytoplasmic vacuolizations shown repeatedly in parenchymal cells of both acini and ducts. Disturbance in cellular metabolism and sodium ions entering the cell have also been reported to cause this damage. This osmotic effect initiates degradation of large macromolecules within the injured cell and leads to the presence of cytoplasmic vacuoles. Furthermore, other cytoplasmic vacuolations might be due to degeneration of other cell organelles such as Golgi apparatus which appear as empty spaces ( Ankily *et al*., 2020).

In our study, the submandibular salivary glands of rats injected with EGF after DXR presented great improvement in the gland architecture. Resolution of vacuolar degeneration was noted apart from minute areas of microvesicles detected in the parenchymal tissue. The acini and ducts cells were more alike to those of the control group. The anti-inflammatory response of EGF was evident in this current work and has been confirmed in previously conducted studies. Berlanga *et al*. (2002) recorded a protective effect of EGF on the intestine from ischemia/reperfusion injury. They noticed the marked reduction of inflammatory infiltrates (mainly neutrophils) in the intestinal tissue after EGF injection. They also registered decreased level of TNF-α (a major pro-inflammatory cytokine), which may contribute to the cytoprotective effect.

The healing potential of EGF was documented in several studies. It was prevalent in epithelial cell re-epithelialization, proliferation, migration and renewal of gastric glands during renal epithelium regeneration ( Flaquer *et al*., 2010), gastric ulcer healing ( Tarnawski & Ahluwalia, 2012), corneal epithelium ( Jeon *et al*., 2018) and salivary glands ( Al-Ankily *et al*., 2018; Shamel *et al*., 2017).

EGF binding to EGF receptor results in auto phosphorylation of receptor tyrosine kinase and activation of signal transduction pathways that are included in the modulation of cellular division, differentiation and persistence. EGF assists epithelial cell regeneration and plays a vital role in dermal wound healing through motivation of proliferation and migration of keratinocytes ( Zeng & Harris, 2014).

Ki-67 is localized during active phases of the cell cycle and its expression is used as a sign of proliferation rate ( Faur *et al*., 2015). It is found in all multiplying cells (normal and up-normal cells) and has been shown to be an admirable proliferation marker to detect the growth rate of certain cell populations. It is also used as the proliferation indicator to evaluate several categories of tumors ( Brown & Gatter, 2002; Do Prado *et al*., 2011; Tadbir *et al*., 2012).

Dayan *et al*. (2002) examined time related alterations in the proliferative capacity of parenchymal cells of human labial salivary gland using Ki-67 as a proliferative marker. They reported positive immuno-expression of Ki-67 within the acini and ducts of the glands in all groups. These findings were in agreement with our results as mild cytoplasmic immunoreactivity for Ki-67 protein was registered in the submandibular salivary gland tissue of the control group. Furthermore, it has been considered that Ki-67 protein localization are not binary as it is continuously decreased during G0 and G1 and is continuously increased from the start of S phase till mitotic exit ( Miller *et al*., 2018).

Nonetheless, Birajdar *et al*. (2014) found Ki‑67 expression in all cases of normal oral epithelium to be mainly presented in the parabasal layer where the numbers of proliferating cells were limited in comparison with the basal cell layer. Furthermore, Hagiwara *et al*. (2013) detected Ki-67 mainly in the parabasal cells and infrequently in the basal cells in the normal squamous epithelium.

Ki-67 proliferation index was found prominently reduced following chemotherapy treatment, showing the anti-proliferative effect on tumors ( Miller *et al*., 2003 and Miller *et al*., 2006; Lee *et al*., 2008). An unexpected finding in the current study was the significant increase in the expression of Ki-67 in the submandibular salivary glands of rats, particularly the secretory terminal portions, secondary to DXR in comparison with control group (p<0.01).

Sasaki *et al*. (1987) studied Ki-67 in HeLa S3 cells (human cell line derived from cervical cancer cells), and showed an increase in Ki-67 antigen after treatment with DXR, as well as its continuous expression throughout the cell cycle. They hypothesized that this is due to the maintaining response of Ki-67 antigen in the cell cycle; interference in DNA replication might cause a reactive enhancement of the Ki-67 protein.

Kausch *et al*. (2003) found that the expression of Ki-67 is increased at G2/M, which is exactly the period during which DXR induces apoptosis. They suggested that DXR could have an inhibitory effect on Ki-67 protein production, which may induce apoptosis. However, cancer cells, in an attempt to survive this effect, increase their mRNA to produce more protein. Ultimately, the production of protein by the cell and induction of apoptosis by DXR reaches an equilibrium, the result of which has been a lack of change in the protein after DXR treatment.

These results were also constant with findings in human hepatocellular carcinoma in which DXR treatment caused the acceleration of cell cycle transition; at an early time point allowing cell cycle continuance, but finally leading to cell cycle arrest ( Choi *et al*., 2012).

According to Etemad-Moghadam *et al*. (2013) a significant increase occurred in Ki-67 mRNA following incubation of cancer cells with DXR, but there was no change in the expression of its protein. However, they failed to explain the exact function and role of Ki-67 in proliferation and cell cycle.

Chemotherapy targets rapidly proliferating cells that are closest to blood vessels but poorly penetrated tumor cells located distal to functional blood vessels (hypoxic regions). Hypoxic cells do not respond to treatment because of the cytotoxic effects generated by oxygen-dependent free radicals. Surviving hypoxic cells in intervals between treatments might re-oxygenate and proliferate from enhanced supply of nutrients released from digestion of dead cells close to the blood vessels. Saggar & Tannok (2015) noted that DXR resulted in the highest increase in Ki-67 cells in reoxygenated surviving hypoxic cells.

Hultman *et al*. (2018) found that post DXR treatment the BE (2)-C (neuroblastoma cell line derived from human bone marrow) tumor growth presented a remarkable increase in Ki-67-index (from 43% to 64%; p<0.01), thus indicating a move towards cycling cells by application of DXR. Tredan *et al*. (2007) previously determined the same hypothesis and found *in vitro* that quiescent (G0) tumor cells enter cell cycle after DXR treatment.

Unexpectedly, we found that Ki-67 proliferation marker expression decreased significantly in the DXR+EGF group in comparison to the DXR group (p<0.001). Weak to mild cytoplasmic immunoreactivity for Ki-67 protein was shown in the parenchymal tissue of the glands of the EGF supplemented group in a manner matching its expression in the control group. Although the expression was a bit stronger than the control, the statistical correlation was not significant (P>0.05). The immunohistochemical results might be correlated with the ultrastructure findings, as binucleation was frequently encountered in DXR group displaying high expression of the proliferation marker, while being unidentified with EGF treatment of low antigen expression ( Mansy *et al*., 2020).

Comparable findings were reported by Fatimah *et al*. (2012), as they found EGF significantly decreased the pluripotent genes expression of cultured human amnion epithelial cells. It is likely that the mitogenic EGF did not favor abnormal proliferation, as had been unexpectedly detected in the current investigation secondary to DXR. In a previous study, a disruption in normal expression of EGF was found correlated with improved proliferation and differentiation of medial cells in developing palate and resulted in cleft palate in rat embryo ( Abbott & Bimbaum, 1990).

## Conclusions

We conclude that EGF has a cytoprotective and reparative effect against DXR induced changes on salivary gland tissue in rats. DXR injection significantly increased Ki-67 immunoexpression in the glandular tissue. However, exogenous EGF preserved the immunohistochemical expression of Ki-67 in the glands or restored it to approximately to the normal level.

## Data availability

### Underlying data

Figshare: The Role of Exogenous EGF on Ki-67 proliferation marker expression on Submandibular Salivary Gland of Albino Rats Receiving Doxorubicin, https://doi.org/10.6084/m9.figshare.13042625.v4 ( Shamel, 2020).

This project contains the following underlying data:

- Original, unedited light microscopy images in TIFF format
- Original, unedited Ki67 immuno-expression images in TIFF format
- Area percentage of Ki-67 immuno-expression for all 21 rats in excel sheet

Data are available under the terms of the Creative Commons Attribution 4.0 International license (CC-BY 4.0).

## References

[ref-1] Abbott BD Bimbaum LS: TCDD-induced altered expression of growth factors may have a role in producing cleft palate and enhancing the incidence of clefts after coadministration of retinoic acid and TCDD. *Toxicol Appl Pharmacol.* 1990;106(3):418–432. 10.1016/0041-008x(90)90337-t 2260090

[ref-2] Al-Ankily MM Shamel M Bakr M: Epidermal growth factor restores cytokeratin expression in rats with diabetes. *J Res Med Dent Sci.* 2018;6(1):196–203. Reference Source

[ref-3] Ankily M Shamel M Bakr M: Epidermal Growth Factor Improves the Ultrastructure of Submandibular Salivary Glands of Streptozotocin Induced Diabetic Rats - A Qualitative Study. *International Journal of Medical and Dental Sciences.* 2020;9(1). 10.18311/ijmds/2020/24452

[ref-4] Ayla S Seckin I Tanriverdi G: Doxorubicin Induced Nephrotoxicity: Protective Effect of Nicotinamide. *Int J Cell Biol.* 2011;2011:390238. 10.1155/2011/390238 21789041 PMC3140777

[ref-5] Berlanga J Prats P Remirez D: Prophylactic use of Epidermal Growth Factor Reduces Ischemia/reperfusion Intestinal Damage. *Am J Pathol.* 2002;161(2):373–9. 10.1016/S0002-9440(10)64192-2 12163361 PMC1850750

[ref-6] Bielack SS Erttmann R Kempf-Bielack B: Impact of scheduling on toxicity and clinical efficacy of doxorubicin: What do we know in the mid-nineties? *Eur J Cancer.* 1996;32A(10):1652–1660. 10.1016/0959-8049(96)00177-3 8983270

[ref-7] Birajdar SS Radhika MB Paremala K: Expression of Ki-67 in normal oral epithelium, leukoplakic oral epithelium and oral squamous cell carcinoma. *J Oral Maxillofac Pathol.* 2014;18(2):169–176. 10.4103/0973-029X.140729 25328294 PMC4196282

[ref-8] Brown DC Gatter KC: Ki67 protein: the immaculate deception? *Histopathology.* 2002;40(1):2–11. 10.1046/j.1365-2559.2002.01343.x 11903593

[ref-9] Choi SY Shen YN Woo SR: Mitomycin C and doxorubicin elicit conflicting signals by causing accumulation of cyclin E prior to p21WAF1/CIP1 elevation in human hepatocellular carcinoma cells. *Int J Oncol.* 2012;40(1):277–86. 10.3892/ijo.2011.1184 21887464

[ref-10] Dayan D Vered M Sivor S: Age-related changes in proliferative markers in labial salivary glands: a study of argyrophilic nucleolar organizer regions (AgNORs) and Ki-67. *Exp Gerontol.* 2002;37(6):841–850. 10.1016/s0531-5565(02)00019-0 12175484

[ref-11] do Prado RF da Silva Machado AL Dias Colombo CE: Immunohistochemical study of the expression of fatty acid synthase and Ki-67 in salivary gland tumors. *J Oral Pathol Med.* 2011;40(6):467–475. 10.1111/j.1600-0714.2011.01023.x 21385214

[ref-12] Etemad-Moghadam S Fouladde S Azizi E: *In vitro* study on the effect of doxorubicin on the proliferation markers MCM3 and Ki-67. *J BUON.* 2013;18(4):1062–1068. 24344040

[ref-13] Fatimah SS Tan GC Chua KH: Effects of epidermal growth factor on the proliferation and cell cycle regulation of cultured human amnion epithelial cells. *J Biosci Bioeng.* 2012;114(2):220–7. 10.1016/j.jbiosc.2012.03.021 22578596

[ref-14] Faul F Erdfelder E Buchner A: Statistical power analyses using G*Power 3.1: Tests for correlation and regression analyses. *Behav Res Methods.* 2009;41(4):1149–1160. 10.3758/BRM.41.4.1149 19897823

[ref-15] Faur AC Sas I Motoc AGM: Ki-67 and p53 immunostaining assessment of proliferative activity in salivary tumors. *Rom J Morphol Embryol.* 2015;56(4):1429–1439. 26743291

[ref-16] Flaquer M Romagnant P Cruzado JM: Growth factors and renal regeneration. *Nefrologia.* 2010;30(4):385–93. 10.3265/Nefrologia.pre2010.Jun.10463 20651879

[ref-17] Ghosh J Das J Manna P: The protective role of arjunolic acid against doxorubicin induced intracellular ROS dependent JNK-p38 and p53-mediated cardiac apoptosis. *Biomaterials.* 2011;32:4857– 4866. 10.1016/j.biomaterials.2011.03.048 21486680

[ref-18] Hagiwara S Yamamoto N Furue H: Pathological analysis of Ki-67 and CD109 expression in tongue squamous cell carcinoma. *J Oral Maxillofac Surg Med Pathol.* 2013;25(3):276–281. 10.1016/j.ajoms.2012.10.002

[ref-19] Hofmann K Bucher P: The FHA domain: a putative nuclear signalling domain found in protein kinases and transcription factors. *Trends Biochem Sci.* 1995;20(9):347–349. 10.1016/s0968-0004(00)89072-6 7482699

[ref-20] Hultman I Haeggblom L Rognmo I: Doxorubicin-provoked increase of mitotic activity and concomitant drain of G0-pool in therapy-resistant BE(2)-C neuroblastoma. *PLoS One.* 2018;13(1):e0190970. 10.1371/journal.pone.0190970 29342186 PMC5771584

[ref-21] Jensen SB Pedersen AM Reibel J: Xerostomia and hypofunction of the salivary glands in cancer therapy. *Support Care Cancer.* 2002;11(4):207–225. 10.1007/s00520-002-0407-7 12673459

[ref-22] Jeon S Choi SH Wolosin JM: Regeneration of the corneal epithelium with conjunctival epithelial equivalents generated in serum- and feeder-cell-free media. *Tissue Eng Regen Med.* 2018;15(3):321–332. 24357922 PMC3867160

[ref-23] Karaman A Fadillioglu E Turkmen E: Protective effects of leflunomide against ischemia-reperfusion injury of the rat liver. *Pediatr Surg Int.* 2006;22(5):428–434. 10.1007/s00383-006-1668-x 16555109

[ref-24] Kausch I Lingnau A Endl E: Antisense treatment against Ki-67 mRNA inhibits proliferation and tumor growth *in vitro* and *in vivo*. *Int J Cancer.* 2003;105(5):710–716. 10.1002/ijc.11111 12740923

[ref-25] Lee J Im YH Lee SH: Evaluation of ER and Ki-67 proliferation index as prognostic factors for survival following neoadjuvant chemotherapy with doxorubicin/docetaxel for locally advanced breast cancer. *Cancer Chemother Pharmacol.* 2008;61(4):569–77. 10.1007/s00280-007-0506-8 17508214

[ref-26] Mansy M Malak S Mubarak RT: The Effect of EGF on the Ultrastructure of Submandibular Salivary Glands of Albino Rats Receiving Doxorubicin. *Annals of Dental Specialty.* 2020;8(1):26–33. Reference Source

[ref-28] Miller I Min M Yang C: Ki67 is a Graded Rather than a Binary Marker of Proliferation versus Quiescence. *Cell Press.* 2018;24(5):1105–1112.e5. 10.1016/j.celrep.2018.06.110 30067968 PMC6108547

[ref-29] Miller WR Dixon JM Macfarlane L: Pathological features of breast cancer response following neoadjuvant treatment with either letrozole or tamoxifen. *Eur J Cancer.* 2003;39(4):462–468. 10.1016/s0959-8049(02)00600-7 12751376

[ref-30] Miller WR White S Dixon JM: Proliferation, steroid receptors and clinical/pathological response in breast cancer treated with letrozole. *Br J Cancer.* 2006;94(7):1051–1056. 10.1038/sj.bjc.6603001 16538221 PMC2361236

[ref-31] Ohlsson B Jansen C Ihse I: Epidermal Growth factor Induces Cell Proliferation in Mouse Pancreas and Salivary glands. *Pancreas.*Department of Surgery, University Hospital. Lund. Sweden.1997;14(1):94–98. 10.1097/00006676-199701000-00014 8981513

[ref-32] Osikov MV Telesheva LF Likhacheva AG: Effect of Local Application of Epidermal Growth Factor on Innate Immunity and Cell Composition of Destruction Focus in Experimental Thermal Injury. *Bull Exp Biol Med.* 2014;157(3):307–10. 10.1007/s10517-014-2552-7 25065306

[ref-33] Parment K Zetterberg A Ernerudh J: Long-term immunosuppression in burned patients assessed by *in vitro* neutrophil oxidative burst (PhagoburstW). *Burns.* 2007;33(7):865–871. 10.1016/j.burns.2006.11.011 17537580

[ref-34] Plaat B Kole A Mastik M: Protein synthesis rate measured with L-[1– ^11^C] tyrosine positron emission tomography correlates with mitotic activity and MIB-1 antibody-detected proliferation in human soft tissue sarcomas. *Eur J Nucl Med.* 1999;26(4):328–332. 10.1007/s002590050394 10199937

[ref-35] Saad SY Najjar TA Al-Rikabi AC: The preventive role of deferoxamine against acute doxorubicin-induced cardiac, renal and hepatic toxicity in rats. *Pharmacol Res.* 2001;43(3):211–218. 10.1006/phrs.2000.0769 11401411

[ref-36] Saggar JK Tannock IF: Chemotherapy rescues hypoxic tumor cells and induces their reoxygenation and repopulation - an effect that is inhibited by the hypoxia-activated pro-drug TH-302. *Cancer Sci.* 2015;106(10):1438–47. 25677696 10.1158/1078-0432.CCR-14-2298

[ref-37] Sasaki K Murakami T Kawasaki M: The cell cycle associated change of the Ki-67 reactive nuclear antigen expression. *J Cell Physiol.* 1987;133(3):579–584. 10.1002/jcp.1041330321 3121642

[ref-38] Schluter C Duchrow M Wohlenberg C: The cell proliferation-associated antigen of antibody Ki-67: a very large, ubiquitous nuclear protein with numerous repeated elements, representing a new kind of cell cycle maintaining proteins. *J Cell Biol.* 1993;123(3):513–522. 10.1083/jcb.123.3.513 8227122 PMC2200129

[ref-39] Shamel M: The Role of Exogenous EGF on Ki-67 proliferation marker expression on Submandibular Salivary Gland of Albino Rats Receiving Doxorubicin. *figshare.*Figure.2020 10.6084/m9.figshare.13042625.v4 PMC7797936 33456767

[ref-40] Shamel M Ankily M Bakr M: Epidermal growth factor restores normal levels of myosin expression in submandibular salivary glands of rats treated with botulinum toxin. *Journal of Advanced Medical and Dental Sciences Research.* 2017;5(1):5–9. Reference Source

[ref-41] Shi SR Liu C Young L: Development of an optimal antigen retrieval protocol for immunohistochemistry of retinoblastoma protein (pRB) in formalin fixed, paraffin sections based on comparison of different methods. *Biotech Histochem.* 2007;82(6):301–309. 10.1080/10520290701791763 18097796

[ref-42] Sobecki M Mrouj K Colinge J: Cell-Cycle Regulation Accounts for Variability in Ki-67 Expression Levels. *Cancer Res.* 2017;77(10):2722–34. 10.1158/0008-5472.CAN-16-0707 28283655

[ref-43] Tadbir AA Pardis S Ashkavandi ZJ: Expression of Ki67 and CD105 as proliferation and angiogenesis markers in salivary gland tumors. *Asian Pac J Cancer Prev.* 2012;13(10):5155– 5159. 10.7314/apjcp.2012.13.10.5155 23244127

[ref-44] Tarnawski AS Ahluwalia A: Molecular mechanisms of epithelial regeneration and neovascularization during healing of gastric and esophageal ulcers. *Curr Med Chem.* 2012;19(1):16–27. 10.2174/092986712803414088 22300072

[ref-45] Tredan O Galmarini CM Patel K: Drug resistance and the solid tumor microenvironment. *J Natl Cancer Inst.* 2007;99(19):1441–54. 10.1093/jnci/djm135 17895480

[ref-27] Tretiakova MS Wei W Boyer HD: Prognostic value of Ki67 in localized prostate carcinoma: a multi-institutional study of >1000 prostatectomies. *Prostate Cancer Prostatic Dis.* 2016;19(3):264–270. 10.1038/pcan.2016.12 27136741 PMC5536893

[ref-46] Yerushalmi R Woods R Ravdin PM: Ki67 in breast cancer: prognostic and predictive potential. *Lancet Oncol.* 2010;11(2):174–183. 10.1016/S1470-2045(09)70262-1 20152769

[ref-47] Zeng F Harris RC: Epidermal growth factor, from gene organization to bedside. *Semin Cell Dev Biol.* 2014;28:2–11. 10.1016/j.semcdb.2014.01.011 24513230 PMC4037350

