# Supplementary material for: The role of exogenous epidermal growth factor on Ki-67 proliferation marker expression in the submandibular salivary gland of albino rats receiving doxorubicin
Source: F1000Res. 2020 Dec 3;9:1393. [Version 1] doi: 10.12688/f1000research.27186.1 (PMC7797936; doi:10.12688/f1000research.27186.1)
Supplement: Peer review history

# Peer review: Reviewer response for version 1

**DOI:** 10.5256/f1000research.30035.r76741

**Published:** 2021-01-08

**Authors:** Mahmoud Serag

**Affiliations:** 1 Division of Prosthodontics, Department of General Dentistry, School of Dental Medicine, East Carolina University, Greenville, NC, USA

The research by Mansy *et al. *is an interesting study which examined the effect of Epidermal growth factor on salivary gland of albino rats receiving doxorubicin detecting ki67 expression as a proliferation marker. 

 The title of the study is suffiecient and reflects the purpose of the study. The methods sections was more than sufficient and I think there is more than enough details that it can be shortened and only referencing is enough. Histological and immunohistochemical results figures were clear.  The manuscript is structurally organized and well written but needed minor modifications in some areas as in the discussion which needed some correlation between other studies and the current study.

 Conclusions were drawn adequately  and the overall manuscript is well constructed and meets the criteria for indexing.

Is the work clearly and accurately presented and does it cite the current literature?

Yes

If applicable, is the statistical analysis and its interpretation appropriate?

Yes

Are all the source data underlying the results available to ensure full reproducibility?

Yes

Is the study design appropriate and is the work technically sound?

Yes

Are the conclusions drawn adequately supported by the results?

Yes

Are sufficient details of methods and analysis provided to allow replication by others?

Yes

Reviewer Expertise:

Dentistry, Oral Health, Prosthodontics

I confirm that I have read this submission and believe that I have an appropriate level of expertise to confirm that it is of an acceptable scientific standard.

# Peer review: Reviewer response for version 1

**DOI:** 10.5256/f1000research.30035.r76739

**Published:** 2021-01-07

**Authors:** Marwa Abdul-Salam Hamied

**Affiliations:** 1 Department of Oral Diagnosis, College of Dentistry, Sulaimani University, Sulaimani, Iraq

The paper by Mansy *et al. *is clearly and accurately presented. They analyzed whether epidermal growth factor effect on ki-67 proliferation marker or not in the submandibular gland parenchyma of albino rats receiving DXR. The submandibular salivary glands of rats injected with EGF after DXR showed great improvement in the glandular tissue. It could be considered a novel treatment for human carcinogenicity and recovery. The title and abstract are appropriate for the content of the text. The figures clearly showed histopathological features and ki-67 expression. Furthermore, the article is well constructed, the experiments were well conducted, and the analysis was well performed.

Is the work clearly and accurately presented and does it cite the current literature?

Yes

If applicable, is the statistical analysis and its interpretation appropriate?

I cannot comment. A qualified statistician is required.

Are all the source data underlying the results available to ensure full reproducibility?

No source data required

Is the study design appropriate and is the work technically sound?

Yes

Are the conclusions drawn adequately supported by the results?

Yes

Are sufficient details of methods and analysis provided to allow replication by others?

Yes

Reviewer Expertise:

Molecular basis of Head and Neck cancers

I confirm that I have read this submission and believe that I have an appropriate level of expertise to confirm that it is of an acceptable scientific standard.

# Peer review: Reviewer response for version 1

**DOI:** 10.5256/f1000research.30035.r75760

**Published:** 2021-01-05

**Authors:** Mushfiq Hassan Shaikh

**Affiliations:** 1 Department of Otolaryngology-Head and Neck Surgery, Schulich School of Medicine & Dentistry, Western University, London, ON, Canada

**Is the study design appropriate and is the work technically sound?**

 Answer: The study design is okay and the work is technically sound. However, reviewer has a concern about the experiment length. The authors have performed the drug experiment on rats for 7 days. Can the authors provide reference of such study length is used in any other previous studies? If so, please add those references in the manuscript.  

**If applicable, is the statistical analysis and its interpretation appropriate?**

 Answer: The statistical analysis and its interpretation was appropriate, however, the reviewer feels that authors should re-plot figure 3 using statistical software. 

**Are the conclusions drawn adequately supported by the results?**

 Answer: The conclusion drawn adequately, however need some addition and modification. the reviewer has discussed this in the report. 

**Reviewer’s Report: **This is an interesting study. Authors in this manuscript presented their study about the role of exogenous epidermal growth factor (EGF) on Ki-67 proliferation marker expression in the submandibular salivary gland on albino rats receiving doxorubicin. To understand the role of EGF in submandibular salivary gland, authors have selected albino rats for this study. The expression pattern of Ki-67 proliferation marker was tested in albino rats treated with doxorubicin, using immunohistochemistry (IHC) technique. The manuscript is structurally organized, however, the writing appears to lack clarity in several places. In the manuscript, especially in the ‘Discussion’ section, the authors have discussed, and referred to different interesting topics, however, in several places failed to correlate those information with the current study. This makes those information irrelevant to mention unless authors make some effort to show the relevance. Therefore, the reviewer feels that rewriting and reorganising the ‘discussion’ section in succinct manner would be needed to enhance the quality of the manuscript. The reviewer has provided some suggestions below for the modification of the ‘discussion’ section. Similar suggestion would apply for the ‘Methods’ section, as the section is unnecessarily too long and in some cases lack proper scientific language use. The authors should consider rewriting the ‘methods’ section in succinct form as well. As the authors have demonstrated changes in the rat tissue after the injection of EGF, which is encouraging, but what does it means, that is, what is the significance of doing this study? The authors should consider adding few sentences about the significance of this study in the manuscript.

 The authors have performed this study on rat’s epithelium, but drawn comparisons with human epithelial cells, which are considered two different species. Have the authors performed investigation and observed similar findings in human tissue? If not, then the authors should carefully conclude the study with their findings and make relevance with the human. Furthermore, the authors have performed the drug experiment on rats for 7 days, the reviewer is concerned about the length of the experiment. Can the authors provide reference of such study length is used in any other previous studies? If so, please add those references in the manuscript.      

 Although this manuscript might have met the criteria for publication on this journal should the following several concerns be addressed properly.

1. The title of the manuscript looks too long, authors should consider meaningful shortening of title.
2. The background of the abstract needs more clarity. Rather than just copying the title here, authors should add two sentences on the background and significance of the study.
3. Under the ‘methods’ section of the abstract, what did the authors mean by ‘immunohistochemical expression of Ki67 sections’? What is this ‘Ki67 section’? Please clarify.
4. Under the ‘results’ section of the abstract, ‘However, Ki-67 expression in the glandular tissue was restored to normal levels’. What did the authors mean by ‘normal levels’? Similar term ‘normal level’ was used in Conclusion. What is the normal level of Ki67 expression? Please, clarify.
5. In the first paragraph of the ‘Introduction’ section, authors mentioned, Ki67 is ‘used for investigations of many cancer types’, can the authors clarify for what sort of investigations? Furthermore, ‘Ki-67 has similar essential properties with the other proteins known to regulate cell cycle’, can the authors explain, what are those essential properties?
6. In the third paragraph of the ‘Introduction’ section, ‘The division activity measured by Ki-67…..’, the authors should explain, what this ‘division activity’ meant here?
7. In the fifth paragraph of the ‘Introduction’ section, the sentence, ‘Apart from xerostomia, 50% of the salivary glands of the patients showed ductal dilation, cyst formation, degenerated acini and inflammatory cell presence.’ Did authors mean those conditions were seen in normal healthy patient or in cancerous patients ? It is confusing here, please clarify.
8. In the sixth paragraph of the ‘Introduction’ section, ‘….. the role of exogenous EGF injection on the Ki-67 immuno-expression in submandibular salivary gland….’ , authors should consider rewriting this sentence, as this sounds confusing, especially, it sounds like role of EGF injection on Ki-67 expression.
9. In the first paragraph of the ‘Methods’ section, authors mentioned that they have followed institutional guidelines for animal study. Can the authors mention in detail what guideline their institution follows, is it Declaration of Helsinki guidelines ? If so, please mention that.
10. In the fourth paragraph of the ‘methods’ section, did the authors kept all 21 rats in one case of separate cages ? Please, clarify.
11. The authors should consider rewriting the ‘methods’ section in succinct but mention all necessary details, as this section is unnecessarily too long and lacks proper scientific language use.
12. Many places across the manuscript, authors used the term ‘Ki67-immuno expression’. Please use, just Ki-67 expression, which will be sufficient and sounds better. Furthermore, authors should use a consistent term throughout the manuscript not interchange between ‘Ki-67 immuno expressions’ and ‘Ki-67 immuno reactivity’, as it becomes confusing. For example, in the fourth paragraph of the ‘results section’, authors mentioned Ki-67 immuno-expression, but in Figure 3, it was Ki-67 immuno-reactivity.
13. In the 2nd paragraph of the ‘Results’ section, ‘DXR+EGF’ group (group III) sections showed great enhancement in the structural features of the gland’, what did the authors mean by ‘enhancement in the structural features of the gland’ here ? Were there increment in the number of structures or enlargement of structures ? Please, clarify.     
14. In the 4th paragraph of the ‘Results’ section, ‘DXR was found to increase the immune-expression of Ki-67 protein in the submandibular salivary gland……..’ What did the author mean in this sentence, is not clear? Did the authors mean DXR increased the expression of Ki-67 ? If so, how or by which mechanism?
15. The authors should consider to remake figure 3, as this, at current state, does not provide much of information and rather confusing. Is the control here indicate 100% ? If so, what is Area % less than 8 ? Y axis numbers or percentages are confusing. The reviewer suggest the authors could use statistical programs such as, Graph Pad Prism, SPSS or R programming to plot this figure properly. Typically, Microsoft Excel is not considered as an ideal stat platform for publication. Furthermore, please add figure legend after title of the figure for Figure 3.
16. In table 1, authors mentioned ‘Area percentage’ but inside the table no percentage sign. Please, add percentage sign where appropriate.
17. In the 5th paragraph of the ‘Results’ section, it reads, ‘increase in immune-reactivity to the proliferation antigen….’. Can authors clarify which ‘Proliferation antigen’ they are referring here ?
18. Many places across the manuscript, authors used ‘not significant (p>0.05)’. Use of this form is a bit confusing while reading, therefore, reviewer suggests the author should only use p-value across the text if anything is significant, such as, (p<0.05). Alternatively, mention the exact p-value, in case of non-significant cases.
19. Under the ‘Discussion’ section, 4 ^th^, 5 ^th^ and 6 ^th^ paragraphs do not fit well in discussion section, authors may consider moving them to introduction section but in more briefly manner.
20. The paragraph 8 of the discussion section, authors referred about the Ki-67 expression in normal oral epithelium. How the authors justify the relevance with the current study providing the above information in this paragraph?
21. The paragraph 9 and 10 in the discussion section should be merged as one paragraph.
22. In paragraph 12 of the discussion section, authors starts the paragraph with ‘These results were also consistent….’. What did the authors mean by ‘These results’ here? Which results authors referring to? Please, clarify as the whole sentence is unclear.
23. In paragraph 13 of the discussion section, authors talked about Ki-67 miRNA expression in another study. How the authors justify the relevance with the current study providing the above information in this paragraph?
24. In paragraph 14 of the discussion section, ‘Chemotherapy targets rapidly……functional blood vessel’ , this sentence needs reference. Please, add reference.
25. In paragraph 17 of the discussion section, authors highlights the EGF effects in pluripotent gene expression. Please, justify the relevance of the above mentioned topic with the current study.
26. In Conclusion, please write in few sentences about the justification and significance of this current study.

Is the work clearly and accurately presented and does it cite the current literature?

Yes

If applicable, is the statistical analysis and its interpretation appropriate?

Partly

Are all the source data underlying the results available to ensure full reproducibility?

Yes

Is the study design appropriate and is the work technically sound?

Partly

Are the conclusions drawn adequately supported by the results?

Partly

Are sufficient details of methods and analysis provided to allow replication by others?

Yes

Reviewer Expertise:

Head and Neck Cancer, Molecular Biology, Immunotherapy, Genome editing, Cancer genomics, Translational research.

I confirm that I have read this submission and believe that I have an appropriate level of expertise to confirm that it is of an acceptable scientific standard, however I have significant reservations, as outlined above.
